# Targeting cellular senescence in senile osteoporosis: therapeutic potential of traditional Chinese medicine

**DOI:** 10.3389/fmed.2023.1288993

**Published:** 2023-11-24

**Authors:** Yingyi Zhang, Xinfeng Yu, Chengcong Zhou, Keqi Fu, Huan Luo, Chengliang Wu

**Affiliations:** ^1^Institute of Orthopaedics and Traumatology, The First Affiliated Hospital of Zhejiang Chinese Medical University (Zhejiang Provincial Hospital of Traditional Chinese Medicine), Hangzhou, China; ^2^The Third Clinical Medical College, Zhejiang Chinese Medical University, Hangzhou, China; ^3^Sanmen People's Hospital, Taizhou, China; ^4^The First Clinical Medical College, Zhejiang Chinese Medical University, Zhejiang, China; ^5^Department of Physical Education, Zhejiang Chinese Medical University, Zhejiang, China; ^6^Department of Pharmacy, The Second Affiliated Hospital, Zhejiang University School of Medicine, Hangzhou, China

**Keywords:** senile osteoporosis, traditional Chinese medicine, cellular senescence, anti-senescence, therapeutic efficacy

## Abstract

Senile osteoporosis (SOP) is a prevalent manifestation of age-related bone disorders, resulting from the dysregulation between osteoblast (OB)-mediated bone formation and osteoclast (OC)-mediated bone resorption, coupled with the escalating burden of cellular senescence. Traditional Chinese medicine (TCM) herbs, renowned for their remarkable attributes encompassing excellent tolerability, low toxicity, heightened efficacy, and minimal adverse reactions, have gained considerable traction in OP treatment. Emerging evidence substantiates the therapeutic benefits of various TCM formulations and their active constituents, including Zuogui wan, *Fructus Ligustri Lucidi*, and Resveratrol, in targeting cellular senescence to address SOP. However, a comprehensive review focusing on the therapeutic efficacy of TCM against SOP, with a particular emphasis on senescence, is currently lacking. In this review, we illuminate the pivotal involvement of cellular senescence in SOP and present a comprehensive exploration of TCM formulations and their active ingredients derived from TCM, delineating their potential in SOP treatment through their anti-senescence properties. Notably, we highlight their profound effects on distinct aging models that simulate SOP and various senescence characteristics. Finally, we provide a forward-looking discussion on utilizing TCM as a strategy for targeting cellular senescence and advancing SOP treatment. Our objective is to contribute to the unveiling of safer and more efficacious therapeutic agents for managing SOP.

## Introduction

Osteoporosis (OP) is a prevalent metabolic bone disorder that affects individuals worldwide with a substantial incidence rate, primarily characterized by reduced bone mass, heightened bone fragility, and deterioration of microstructural bone tissues (1). Given its high prevalence among the elderly population, senile osteoporosis (SOP) has emerged as a significant global health concern (2). In China, the incidence of OP has risen dramatically, affecting approximately 110 million individuals in the past four decades (3). This prevalent condition poses a significant health risk to the elderly population, necessitating extensive resources for treatment, nursing, and management of SOP, osteoporotic fractures, and associated complications. Consequently, it places a substantial burden on families and society, both in terms of labor and financial resources. Beyond the imbalance between osteoblast (OB)-mediated bone formation and osteoclast (OC)-mediated bone resorption, research findings indicate that the aging process leads to a disproportionate differentiation of bone marrow mesenchymal stem cells (BMSCs) into adipocytes rather than OB. Furthermore, these cells undergo senescence, resulting in bone loss and contributing to the SOP development (4, 5). Hence, targeting fundamental aging mechanisms such as senescence represents a promising avenue for SOP treatment. Although several medications, such as teriparatide, risedronate, and romosozumab, are currently available for SOP treatment, their limited effectiveness is hampered by potential side effects (6–8).

Traditional Chinese medicine (TCM) has garnered wide utilization for the treatment of various diseases, including SOP, due to its minimal adverse effects (9–12). Recent basic research has discovered the anti-senescence effects of numerous TCM formulations (such as the Yiqi Huayu decoction, Bazi Bushen formulations) and their active ingredients (Ginsenoside Rb2) (13–16). Moreover, emerging evidence demonstrates the applicability of TCM and its derivative compounds in treating SOP through their anti-senescence effects (17–19). Nonetheless, a comprehensive review encompassing the treatment of OP, particularly SOP, using TCM with a focus on targeting cellular senescence is currently lacking. To comprehensively understand the anti-senescence mechanisms by which TCM exerts its therapeutic effects on SOP, it is essential to gather evidence from both *in vivo* and *in vitro* studies. Firstly, it is necessary to elucidate the effects of TCM on various animal models of SOP induced by aging to gain insights into its therapeutic potential. Furthermore, after demonstrating the therapeutic effects of TCM on SOP induced by aging *in vivo*, it is essential to delve deeper into the regulatory effects of TCM on senescence characteristics during the treatment of SOP *in vitro*.

Within this review, we delineate the significance of cellular senescence in the context of SOP and provide a comprehensive overview of recent advancements involving TCM formulations and active ingredients derived from TCM that can be harnessed to treat SOP by leveraging their anti-senescence property. Specifically, we highlight the profound effects of TCM on different aging models employed to simulate SOP. Additionally, we recapitulate the therapeutic effect of TCM on SOP by modulating various senescence characteristics, such as oxidative stress, p53, p21, p16, and the senescence of BMSCs. Finally, we present prospects for SOP treatment using TCM by targeting cellular senescence and developing potential therapeutic drugs.

## Cellular senescence in SOP

**Figure 1:**
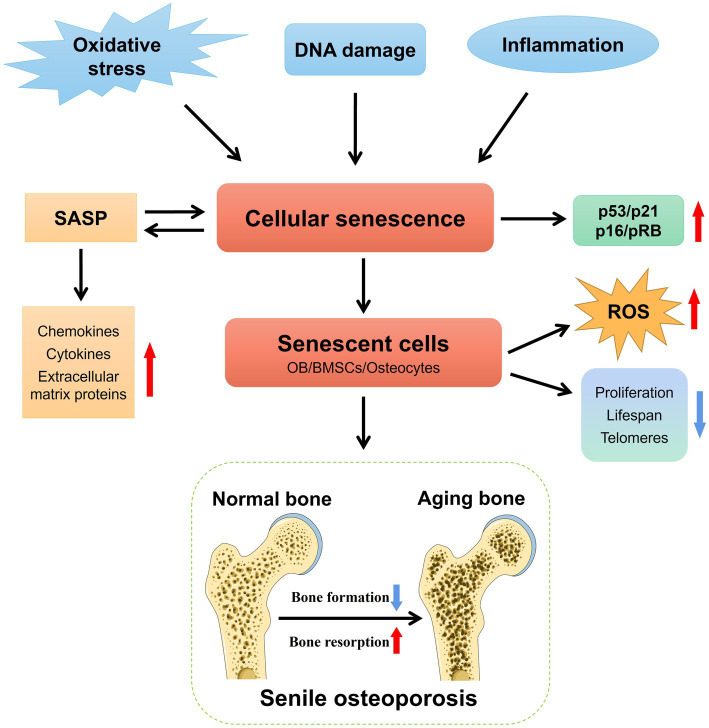
The mechanism underlying cellular senescence-induced SOP. The senescence of OB, BMSCs, and osteocytes plays a crucial role in the pathogenesis of SOP. Various interconnected factors, including heightened oxidative stress, DNA damage, chronic inflammation, and SASP, actively participated in the induction and progression of bone cell senescence in SOP. Red arrows indicate upregulated levels of these molecule entities, while blue arrows denote downregulated levels.

Cellular senescence is a vital process of irreversible cell cycle arrest that is instrumental in tissue remodeling during development and post-injury (20). It occurs in response to various stresses, resulting in DNA damage and the secretion of chemokines, cytokines, and extracellular matrix proteins, which creates a detrimental microenvironment known as senescence-associated secretory phenotype (SASP) (21). Besides, activation of the p53/p21 and p16/pRB tumor suppressor pathways assumes a central role in cellular senescence (22). These pathways, recognized as core markers of senescence, have been observed not only in aged mice but also in aged human bones (23). Accumulation of senescent cells accompanies the aging process and has been linked to the promotion of various age-related diseases, including SOP (24–26). Notably, Farr et al. demonstrated that eliminating senescent cells can prevent age-related bone loss in mice (26). Thus, cellular senescence plays a critical role in the development of SOP. The mechanism through which cellular senescence induces SOP is shown in Figure 1.

Since the development of OP is thought to be caused by increased OC activity, decreased OB activity, compromised osteocyte viability, and reduced osteogenic potential of BMSCs (27–29), the senescence of dysfunctional OB, BMSCs, and osteocytes, significantly impact the pathological progression of SOP (30–33). These senescent cells display senescent-like characteristics such as impaired proliferation, reduced lifespan, shortened telomeres, and increased production of reactive oxygen species (ROS). Given the close relationship between cellular senescence and aging-related diseases, including SOP, various animal models of aging, such as D-galactose (D-gal)-induced mice and senescence-accelerated mice/prone (SAMP) mice, have been established to elucidate the mechanisms underlying cellular senescence in SOP (34, 35).

Recent evidence has shed light on the significant relevance of senescent OB, BMSCs, and components of the SASP to SOP. D-gal has been found to trigger OB senescence through excessive nonmetabolized D-galactitol accumulation, leading to bone senescence and SOP (36). In p21-expressing senescent OB, upregulated HIF-2α expression hinders OB differentiation and function. On the other hand, upregulated HIF-2α expression in OC promotes the transcription of p16 and p21 by directly binding to hypoxia-responsive elements. This interplay between HIF-2α and p16/p21 in senescent OB and OC contributes to age-related bone loss and SOP (37). Studies also have shown that estrogen deficiency could induce bone loss and promote OB senescence, while estrogen administration effectively alleviates the senescence of OB, preserves their function, and accelerates p53 degradation in osteoblastic MC3T3-E1 cells (38–40). Additionally, senescent OB can modulate the function of endothelial cells, induce cellular senescence and apoptosis, and hinder cell proliferation through the exosomal pathway, thus affecting the bone environment during aging through the alterations of angiogenesis (41). The acquisition of SASP in both osteogenic and myeloid lineage cells may contribute to the accelerated skeletal aging phenotype characterized by impaired osteogenesis (42).

Likewise, Geng et al. demonstrated that increased osteocyte senescence and SASP induced by estrogen deficiency accelerate bone loss (32), highlighting the potential role of senescence in estrogen deficiency-induced SOP. Furthermore, the removal of osteocytes leads to the accumulation of SASP in bone marrow osteoprogenitors and cells within the myeloid lineage, further boosting the OC formation while inhibiting osteogenesis, consequently resulting in an accelerated skeletal aging phenotype accompanied by OP (42).

According to a toxicology study, cadmium-induced cellular senescence of BMSCs through the activation of the NF-κB signaling pathway has been found to substantially impact the osteogenic differentiation and predispose BMSCs to adipogenesis, leading to an observable increase in the number of adipocytes and a decrease in mineralization within the bone marrow in Sprague–Dawley rats (43). Other studies have revealed that increased cellular senescence of BMSCs induced by diverse factors, including high-glucose concentrations, oxidative stress, and inflammation micro-environment, compromises the osteogenic differentiation ability of BMSCs and contributes to SOP (44–47). Furthermore, it has been shown that epigenetic modifications, include DNA methylation, histone modifications, chromatin remodeling and posttranscriptional processing through mRNA and noncoding RNAs, play a significant role in regulating BMSCs senescence (48). For instance, a decrease in the expression of lncRNA-*Bmncr* in senescent BMSCs has been associated with an increase in lipogenesis (49), while the suppression of H3K27me3 at the promoter of *p14* and *p16* leads to the activation of corresponding proteins, further exacerbating BMSCs senescence (50). These findings demonstrate the strong involvement of the epigenetic component in senescence-related OP.

Given the significant influence of senescent cells on bone homeostasis, the therapeutic targeting of cellular senescence in SOP has garnered considerable attention. Research into anti-senescence drugs for SOP has intensified, with substances such as Genistein, Lactoferrin, Resveratrol, and Angiotensin 1–7 being investigated (51–54).

## TCM formulations and bioactive ingredients with anti-senescence properties in different aging models for treating SOP

TCM herbs have diverse properties and therapeutic qualities that have been utilized for the effective management of OP (55). Extensive research has highlighted the significant role of senescence in OP, particularly in the context of SOP, therefore, we compiled a summary of the anti-osteoporotic effects of TCM formulations and their active ingredients by using different aging models including naturally aging model, SAMP mice, and D-gal-induced aging model. These findings enhance our understanding of how TCM regulates senescence during SOP treatment, thereby facilitating further research and practical application in clinical settings.

### Naturally aging model

SOP is known to become increasingly prevalent with aging, so animal models of natural aging serve as valuable tools to simulate the aging process and age-related diseases, including SOP, in the human body. Notably, increasing studies have demonstrated the anti-osteoporotic effects of TCM in animal models of natural aging.

Hu et al. found that administration of Xianzhen Gubao (XZGB) capsules to corticosteroid-induced aging rats for a period of 90 days resulted in a marked increase in trabecular area by suppressing bone resorption and a slight increase in bone formation, and higher doses of XZGB were found to yield greater effects (56). A study using metabolomics investigating Fufang Zhenzhu Tiaozhi (FTZ)'s protective effects against aging-induced OP showed that FTZ can reverse abnormal levels of metabolites associated with phospholipids and arachidonic acid metabolism, as well as energy metabolism in 21-month-old female mice when compared to the aging model group (57). Leptin (LEP), a peptide hormone synthesized and secreted by adipocytes, has been identified as playing a crucial role in the regulation of bone metabolism (58). Recent research has demonstrated that Qing’e Decoction (QED) treatment for 3, 6, and 9 months in naturally aging rats can reduce elevated levels of LEP in the serum, thus helping to regulate bone metabolism, maintain trabecular structure and bone quality, delay bone aging, and prevent OP (59).

*Fructus Ligustri Lucidi* (FLL), derived from the fruit of TCM *Ligustrum lucidum Ait*, is a component of numerous TCM formulations used in OP treatment. The combination administration of *Epimedium leaf* (EF) and FLL for 2 months has demonstrated beneficial effects by enhancing the expression of TGF-β1, BMP2, Wnt5a, and IGF-1 while reducing the increase of bone tissue adipocytes in 15-month-old male rats, thus ameliorating low bone mass associated with aging. These findings suggest that the combination of EF and FLL could serve as a potential alternative for treating SOP (60). In a study involving eight-month-old Kunming female mice, FLL did not show a therapeutic effect on aged mice with OP in terms of femur index, fecal Ca, and BMD value due to irreparable bone damage. Interestingly, it demonstrated a protective effect against OP and reduced damage in a model combining D-gal injection with a low calcium diet-induced SOP (61), highlighting the importance of early intervention in TCM-based SOP treatment. Moreover, preclinical evidence of long-term Allicin treatment in aging male rats (ranging from 13 to 21 months) reveals it can provide protection against SOP and reverse deleterious bone biomechanical features associated with aging through the elevation of both bone formation and bone resorption, as well as an increase in bone mineral density (BMD), indicating Allicin may potentially delay the onset of OP (62). Oleanolic Acid (OA), a pentacyclic triterpenoid compound found in over 1,620 plants and medicinal herbs, has shown the ability to induce osteogenic differentiation of BMSCs (63), and effectively improve bone microarchitecture in aged rats (9-month-old) by improving calcium balance and modulating Vitamin D metabolism, making OA a potential drug candidate for the treatment of SOP (64).

### SAMP mice

SAMP mouse model has been widely recognized as a valuable spontaneous experimental model to study age-related secondary OP (35), with numerous characteristics that resemble SOP in humans, including low peak bone mass and impaired OB formation (65), making them highly suitable for studying the therapeutic effects of various TCM interventions and their active components on SOP.

Zhao et al. found that *Eclipta prostrata* significantly improved bone microstructure in SAMP6 mice by regulating the dynamic balance of bone absorption and formation, while it also markedly increased the abundance of bacteria genera Lactobacillus and Lactococcus, suggesting that targeting gut microbiota might represent a novel treatment approach for SOP (66). Similarly, the combination of *Eucommia ulmoides* leaf water extract (EUL) and *Lactobacillus bulgaricus* (LB) has been proven to improve osteoporotic manifestations in SAMP6 mice, including reduced trabecular bone, increased intertrochanteric space, and decreased BMD. This combination treatment also regulates the diversity of gut microbiota (GM) to promote skeletal health (18). In parallel, a comparative study of SAMP6 mice explored the effects of 3 TCM formulations (Hachimi-jio-gan, Juzen-taiho-to, and Unkei-to) on bone loss in SOP disorder found that Hachimi-jio-gan and Juzen-taiho-to treatment could, respectively, decrease the number of mast cells in the bone marrow and serum parathyroid hormone levels while increasing the amount of bone-forming surface and the bone mass (67). Furthermore, Bu-Gu-Sheng-Sui decoction (BGSSD) accelerated the proliferation and differentiation of BMSCs and improved bone trabecular structure to protect bone mass in SAMP6 mice by stimulating the activity of Alkaline phosphatase (ALP) and increasing the expression of Runx2, suggesting BGSSD may constitute a potential drug for preventing and managing SOP by targeting cellular senescence (68). Another study conducted by Liu et al. evaluated the therapeutic potential of Antrodia camphorate alcohol extract (ACAE) for SOP recovery in SAMP8 mice and demonstrated ACAE could upregulate the level of osteogenic genes such as *RUNX2*, *OCN*, and *OPN in vitro* and inhibit bone loss as well as increase the percentage bone volume, trabecular bone number, and BMD *in vivo*, thereby promoting osteogenesis and preventing SOP (69). Another study on total glycosides and polysaccharides of *Cistanche deserticola* in SAMP6 mice exhibited improvements in decreased bone formation, damaged bone microstructure, and regulation of RANKL, BMP-2, and OPG expression by activating the Wnt/β-catenin signaling pathway (19).

Moreover, Xu et al. revealed that OB from SAMP6 mice exhibited lower cellular development and differentiation activity compared to OB from normal aging mice (SAMR1). The expression of Connective tissue growth factor (CTGF) was also found to be lower in SAMP6 mice. Furthermore, it was demonstrated that Icariin, a flavonoid compound derived from *Epimedium brevicornum*, can activate the BMP signaling pathway and downregulate CTGF expression to enhance BMP-2-induced OB differentiation (70). Intragastrical administration of *Astragalus membranaceus* (AM, also known as Huangqi) to SAMP6 mice could improve the femoral BMD and bone microstructure, elevate the calcium and phosphorus contents, and increase the expression of Klotho, Vitamin D receptor (VDR), and CYP27B1 but decrease the expression of FGF23 and CYP24A1, suggesting the curative effect of AM on spontaneous SOP (3). Resveratrol, an edible polyphenolic phytoalexin from *Veratrum grandiflorum*, enhanced bone formation and counteracted accelerated bone loss in SAMP6 mice. It also improved the osteogenic differentiation of senescent BMSCs from SAMP6 mice through the amelioration of Mitofilin-mediated mitochondrial compromise (71). Orcinol glucoside (OG), derived from the extract of *Curculigo orchioides* Gaertn, could attenuate bone loss by inhibiting the formation and bone resorption activities of OC, thus aiding the prevention of SOP. Mechanistical analysis revealed that OG reduced the high levels of oxidative stress in the SAMP6 mice via the Nrf2/Keap1 and mTOR signaling pathways (72).

### D-gal-induced aging model

D-gal, an aldohexose, facilitates the conversion of aldose and hydroperoxide, leading to the production of ROS (73). D-gal has the ability to induce osseous changes resembling senescence characteristics of natural aging, which may contribute to bone loss and SOP in the aging process (36, 74). Consistent with previous findings, TCM also plays a significant role in treating D-gal-induced aging in mice.

A study conducted by Xu et al. found that treatment with Bajitian Wan (BJTW) effectively mitigated D-gal-induced bone loss in aging mice. This effect was observed through the modulation of ALP, OCN, OPG, and RANKL levels (75), which suggests that BJTW exhibits promising anti-senescence properties and holds promise as a treatment option for SOP.

In the D-gal-induced aging rat model, Canthaxanthin treatment significantly increased the BMD, structural mechanics and biomechanics parameters, as well as bone calcium levels, thereby effectively preventing aging and SOP in the aging model rats injected with D-gal for 5 months (76). Orally administration of diosgenin to D-gal-induced aging rats has been found to significantly increase frame and femur volume while reducing porosity and frame density, suggesting diosgenin could potentially prevent bone loss during aging and provide beneficial effects in SOP (77). Similarly, Peptide-Calcium Chelate derived from Antler (*Cervus elaphus*) improved bone microstructure and alleviated age-related bone loss by enhancing calcium absorption in an aging mouse model (78). Cycloastragenol, an aglycone of astragaloside IV isolated from AM Bunge, could decrease serum bone resorption marker (TRACP), augment bone strength, reduce OC number, and improve bone formation in D-gal-treated rats. The potential mechanism behind these effects may be linked to the Cycloastragenol-induced increase in osteoactivin expression, providing preclinical evidence for its potential as a therapeutic agent in treating SOP (79).

## Potential targets of TCM formulations and bioactive ingredients with anti-senescence properites in treating SOP

Cellular senescence, triggered by factors like oxidative stress and the activation of the p53/p21 and p16/pRB tumor suppressor pathways, plays a central role in SOP. In basic research, several TCM formulations and their active ingredients has demonstrated anti-senescence effects (13–16). To better understand the potential targets of TCM formulations and their bioactive ingredients in treating SOP, we also summarize the experiments focusing on reducing oxidative stress, senescence-associated markers and other characteristics such as BMSCs senescence.

### Oxidative stress

Oxidative stress arises when cells generate an excessive amount of ROS, leading to a pathological process (80). Previous studies have demonstrated that senescent cells have higher ROS levels compared to normal cells, suggesting the involvement of ROS in cellular senescence (81). In addition, oxidative stress is closely associated with OP development (82, 83). Extensive research is now focused on the accumulation of ROS as well as ROS-mediated cellular senescence in SOP, with TCM playing a role in this domain.

As a major isoflavone glycoside extracted from the Chinese herb *Pueraria radix*, Puerarin effectively mitigates bone mass loss by inhibiting osteoclastogenesis and suppressing oxidative stress in bone tissues (84). Zhou et al. found that Resveratrol attenuates BMSCs senescence derived from aging rats and directs BMSCs differentiation towards OB lineage through the activation of AMPK and the down-regulation of ROS production. This research provides new insight into the application of TCM herbs and their active constituents for treating ROS/age-induced OP (85). Similarly, Ginkgolide B (GB), a small natural molecule from *Ginkgo biloba*, exhibits pharmacological activities in aging-related diseases, including SOP. It has been shown to significantly enhance bone mass in mice with aging-induced OP by increasing the OPG-to-RANKL ratio while promoting osteogenesis in aged BMSCs and inhibiting osteoclastogenesis in aged macrophages through ROS reduction. These findings suggest the promising potential of GB for further clinical investigation (86). Astragalus Polysaccharide (AP), derived from a commonly used anti-aging TCM herb AM, has a comparable effect in reducing ROS accumulation. It increases Nanog, Sox2, and Oct4 expression, and inhibits mitochondrial ROS accumulation to counteract the senescence of BMSCs induced by ferric ammonium citrate, ultimately promoting osteogenesis (87). Likewise, FLL reverses the decline in interleukin-2, tumor necrosis factor-alpha, and oxidative stress in the serum of D-gal-induced ICR mice, effectively preventing age-related OP by maintaining bone microstructure and strength and inhibiting bone loss initiation (17). β-amyloid (Aβ) deposition and the resultant oxidative damage are significant contributors to aging diseases such as SOP, whereas *Humulus lupulus* L. Extract (HLE) treatment reduces Aβ deposition in bones of APP/PS1 mice, alleviating oxidative stress and modulating bone metabolism. It improves BMD and bone microstructure while substantially restoring the decreased expression of Nrf2, HO-1, NQO1, FoxO1, and SOD-2 in Aβ-damaged OB, indicating that HLE can alleviate Aβ deposition-induced oxidative stress through the activation of the Nrf2 and FoxO1 pathways, providing evidence for its clinical application in the prevention and treatment of SOP (88).

### p53/p21/p16

Cellular senescence arises from replicative and stress-induced senescence, wherein the activation of p53 and p16 leads to the activation of p21, resulting in cell cycle arrest (89). OP mice and aged bones from humans have recognized p53, p21, and p16 as indicators of senescence (23, 90, 91). Research focused on these senescence markers in the context of OP holds significant potential. Indeed, numerous TCMs and their active constituents are currently under investigation in this area.

Zuogui Wan (ZGW), a classical TCM prescription for senile disorders and anti-aging, has been demonstrated to regulate Wnt signaling and suppress the expression of senescence-related factors such as p53, p21, and p16 to enhance cell proliferation, ameliorate DNA damage, and reduce SASP secretion, thereby maintaining osteogenic differentiation of rat BMSCs (92). Yuan et al. demonstrated that treatment with Si Jun Zi Tang (SJZT), another classical TCM prescription, improved OP in aging mice, as evident from micro-CT analysis results. The protein expression of p53, p21, and p16 were also significantly reduced in SJZT-treated mice (93). Network pharmacology, as an emerging discipline, provides a new network model of “multiple targets, multiple effects, and complex diseases” to explain the mechanism of TCM treatment. Several studies investigating the potential pharmacological process and specific mechanisms of TCM in treating OP (Liuwei Dihuang Pill, *Eucommia ulmoides* cortex, Ursolic acid, Jiawei Buguzhi Pill) have demonstrated that a potential association with p53 (94–98). Nevertheless, these predictions made by network pharmacology require further validation through future *in vivo* and *in vitro* experiments to strengthen the basic research evidence of TCM in the treatment of OP, particularly cellular senescence.

Moreover, treatment of human OB with Resveratrol and Anthocyanins led to a decrease in *p53* mRNA expression and an increase in cell proliferation, thus promoting OB differentiation and reducing RANKL-induced bone resorption. These compounds could also serve as a novel therapy for OP (99). Consistently, treatment with Resveratrol in human BMSCs resulted in a significant reduction in SASP secretion levels, as well as decreased expression of senescence-related genes (*p53*, *p16*, and *p21*) and intracellular ROS levels. Meanwhile, it inhibited adipogenic differentiation of human BMSCs, thereby achieving therapeutic effects for OP (53). Likewise, Echinacoside, a phenylethanoid glycoside isolated from a TCM herb *Herba Cistanches* (100), exerted a protective effect on OB by suppressing p53 expression, suggesting its potential for OP treatment (101).

### Others

In addition to the aforementioned information, TCM formulations and their active constituents can address SOP using alternative approaches, such as improving the senescence of BMSCs and regulating multiple signaling pathways.

Du-Huo-Ji-Sheng-Tang (DHJST) and its active component Ligusticum augment *RUNX2* and *BMP-2* gene expression by activating SMAD1/5/8 and ERK signaling pathways, thereby promoting osteogenic activity in human mesenchymal cells (hMSCs). Meanwhile, they are capable of reducing senescence levels in hMSCs during the aging process (102).

Catalpol, the principal bioactive component of *Rehmannia glutinosa*, not only dose-dependently diminishes the proportion of senescent cells in BMSCs but also enhances their osteogenic differentiation and stimulates bone regeneration by partially activating the Wnt/β-catenin pathway (103). Tanshinone IIA (TSNA), a major active component found in *Salvia miltiorrhiza* Bunge (Danshen), can restore the cellular stemness of BMSCs and reverse aging in BMSCs. Mechanistically, TSNA primarily targets *PHGDH* mRNA, upregulating its levels and reducing the high methylation in the promoter region of PHGDH, thereby exerting anti-aging and anti-osteoporotic effects of TSNA on BMSCs (104). In parallel, AM exerts its effects by modulating the Vitamin D-FGF23-Klotho pathway, effectively reversing the reduced cellular viability and osteogenic capacity observed in senescent BMSCs after the *VDR* gene downregulation. This modulation enhances osteogenic ability and prevents age-related OP (105).

## Conclusion and perspectives

SOP, a prevalent form of OP, represents a significant consequence of age-related bone disorders (106). Nevertheless, the pathogenesis of SOP remains intricate, and the precise mechanisms underlying its onset and progression require further elucidation. As discussed above, cellular senescence and the senescence-associated secretory phenotype (SASP) play a crucial role in various age-related conditions, including SOP. Hence, targeting senescence or eliminating senescent cells holds great promise.

TCM contains numerous bioactive compounds with diverse pharmacological activities. Moreover, when administered *in vivo*, TCM can generate additional bioactive or inactive metabolites. Importantly, TCM formulations, herbs, and their active ingredients, such as EF, FLL, Resveratrol, and ZGW, exhibit significant anti-senescence effects, whose efficacy against SOP has been verified through both *in vitro* and *in vivo* experiments involving naturally aging mice, SAMP mice, and D-gal-induced mice. These findings provide a theoretical basis for the clinical application of TCM in the treatment of SOP. However, current clinical research on TCM primarily focuses on postmenopausal osteoporosis (PMOP) (107, 108), and it does not adequately distinguish or assess the effects on PMOP and SOP, separately, which makes it difficult to gather specific clinical data for SOP treatment. It is important to have more clinical data in the future to establish the effectiveness of TCM in treating SOP. Nevertheless, clinical trials have been conducted on TCM formulations and their active ingredients, such as QED, AM, and Resveratrol, for the treatment of other diseases (109–111). Moreover, preclinical studies have demonstrated the targeting of cellular senescence by these medicines in the context of SOP, as mentioned above, which provides confidence for the initiation of clinical trials on these TCM formulations for SOP treatment.

Apart from SOP, other aging-related diseases, including neurodegenerative diseases, cardiovascular diseases, and metabolic diseases, have been effectively treated with TCM (112–114). Since potential correlations may exist between senescence and the onset of different senescence-related diseases, exploring new therapeutic TCM drugs for treating SOP by targeting cellular senescence can draw inspiration from the treatments of other senescence-related diseases using TCM herbs, formulations, or their active ingredients. Current research has identified senescence as a modifiable through hormones, drugs, and inhibitors such as glucocorticoids, Dasatinib and Quercetin, metformin, SASP inhibitors (rapamycin and ruxolitinib), and senolytics (115–120). To gain further insights into the anti-senescence effects of TCMs and their active ingredients in SOP, combining TCM with proven anti-aging substances and drugs, alongside the aforementioned approaches, in basic research and preclinical trials is possible. Alternatively, conducting comparative studies to evaluate the effects of TCM and the aforementioned drugs (as positive control) separately is another avenue to explore. DNA damage is a crucial factor in maintaining genetic stability and is also implicated in triggering cellular senescence (121), contributing to the development of age-related diseases, including SOP (122). Notably, TCM formulations and their active compounds such as Bazi Bushen, Yifuning, Deoxyschisandrin, and Schisandrin B have shown therapeutic effects on age-related diseases by regulating DNA damage (123–125). Therefore, developing TCM formulations that target DNA damage repair for the treatment of SOP holds promising prospects. As mentioned earlier, epigenetic alterations play a crucial role in both normal bone formation and function, as well as in pathogenesis of SOP (48, 126). Natural TCM compounds like resveratrol, sulforaphane, specific phenolic acids and anthocyanins, have been shown to regulate bone remodeling by influencing the bone epigenome (127). Therefore, conducting further research on the mechanisms of TCM in treating SOP can be achieved through studying epigenetic modifications such as DNA methylation, histone modifications, and post-transcriptional regulation.

It is essential to note that the anti-senescence effects of TCM are not limited to a singular approach. For instance, Resveratrol not only enhances osteogenic differentiation of senescent BMSCs from SAMP6 but also reduces intracellular ROS levels and senescence-related genes (*p53*, *p16*, and *p21*). Consistently, FLL exhibits anti-senescence effects in both naturally aging mice and D-gal-induced aging mice. Furthermore, studies have demonstrated that D-gal can generate ROS (73, 128), which can contribute to the development of SOP. In this context, TCM formulations such as FLL have been shown to possess antioxidant properties when used to treat SOP induced by D-gal in mouse models. Thus, a comprehensive understanding of the anti-senescence effects of TCM in SOP necessitates consideration of different mechanisms of senescence. Noteworthy, when applying different aging models to simulate SOP, it is also necessary to consider their advantages and disadvantages. Naturally aging models can better replicate the gradual aging process of the human body, but they have the drawback of requiring prolonged modeling time. The D-gal-induced aging model is commonly utilized to investigate the pathological features associated with senescence, such as mitochondrial dysfunction, excessive formation of glycation products, and oxidative stress (74), however, additional research is necessary to explore these pathways in the D-gal-induced model and determine its similarity to the natural aging process. SAMP mice exhibit accelerated aging characteristics, allowing researchers to study the pathological manifestations of SOP in a relatively short period, however, they have a shorter lifespan compared to regular mice, which may limit long-term studies on SOP processes, and the accelerated aging phenotype observed in SAMP mice may not fully reflect the complexity and heterogeneity of human aging, potentially limiting the generalizability of findings.

Moreover, numerous studies have indicated that the bone microenvironment associated with senescence enhances the activity of OC progenitors and increases osteoclastogenesis. Interestingly, the elimination of senescent OC progenitors did not affect bone loss in age-related mice, instead, senescent osteocytes have emerged as a critical mechanism in bone aging, suggesting their involvement in age-related bone loss (129). Similarly, senescent immune cells, including macrophages and neutrophils, also contribute to the development of SOP (130). Based on the anti-senescence effects of TCM, it is speculated that TCM also has great potential in treating SOP by targeting senescent OC and immune cells. However, it is crucial to underscore that the clinical significance of TCM in age-related diseases, including SOP, remains incompletely understood. This is partly due to the early stages of research on TCM’s anti-senescence effects and the scarcity of comprehensive and rigorous clinical trials documenting its efficacy. It is worth noting that age is a significant risk factor for another type of OP, PMOP. Research has found that targeting cellular senescence can be a potential approach for PMOP treatment (32, 46, 131). In contrast to SOP, which affects both men and women, PMOP predominantly occurs in women due to decreased ovarian function and a decline in estrogen levels in the body, so the mainstay of prevention of POMP was hormone therapy with estrogen and progestin (HT) or estrogen therapy (ET) (132), TCM has proven to be effective in reducing the risk of OP by its estrogen-like activity (133). Therefore, when using TCM to treat age-related PMOP, the combination of herbs or formulations with estrogenic effects and anti-senescence properties can be considered to enhance the therapeutic effects.

In conclusion, based on evidence from basic research, the utilization of TCM formulations and their active constituents for treating SOP by targeting cellular senescence exhibits promising prospects. However, there is still a lack of clinical data and validation regarding the use of TCM and its active constituents in the context of anti-senescence treatments for SOP. In addition, apart from senescence, the pathological mechanisms of OP also include other factors such as immune dysfunction and inflammation (134, 135). Therefore, further elucidation is warranted to determine whether a specific TCM exerts its anti-osteoporotic effects through a single pathway or multiple pathways, as well as the precise underlying mechanisms. Nevertheless, the mounting evidence strengthens our confidence in the potential of TCM for SOP treatment (Table 1).

**Table 1:** The efficacy of TCM formulations and active constituents in treating SOP by targeting cellular senescence.

Compound	Medicinal materials	Animal model or Cells	Beneficial effects	Reference
N/A	XZGB	Naturally aging model	Increased the trabecular area	(56)
N/A	FTZ		Reversed the abnormal levels of metabolites	(57)
N/A	QED		Reduced the elevated levels of LEP and maintained trabecular structure and bone quality	(59)
N/A	SJZT		Increased the total average BMD	(93)
N/A	*Epimedium* leaf and *Fructus Ligustri Lucidi*		Improved low bone mass by enhancing the expression of TGF-β1, BMP2, Wnt5a, and IGF-1.	(60)
Allicin	*Allium sativum* L.		Reversed deleterious bone biomechanical features and increased BMD	(62)
Oleanolic Acid	N/A		Improved bone micro-architecture through improving calcium balance and modulating vitamin D metabolism	(63, 64)
N/A	BGSSD	SAMP mice	Protected the bone mass and improved bone trabecular structure	(68)
N/A	Hachimi-jio-gan and Juzen-taiho-to		Increased the amount of bone-forming surface and bone mass	(67)
N/A	*Astragalus membranaceus*		Improved the femoral BMD and bone microstructure, elevated the contents of calcium and phosphorus	(3)
N/A	*Eclipta prostrata*		Improved bone micro-structure by regulating the dynamic balance of bone absorption and formation	(66)
*Eucommia ulmoides* leaf water extract	*Eucommia ulmoides*		Increased trabecular bone, BMD, and decreased intertrochanteric space	(18)
*Antrodia camphorata* alcohol extract	*Antrodia camphorata*		Inhibited bone loss and increased the proportion of bone volume, trabecular bone number, and BMD	(69)
Resveratrol	*Veratrum grandiflorum*		Enhanced bone formation and counteracted accelerated bone loss	(71)
Orcinol glucoside	*Curculigo orchioides*		Reduced high levels of oxidative stress	(72)
Total glycosides and polysaccharides of *Cistanche deserticola*	*Cistanche deserticola*		Decreased bone formation and damaged bone microstructure as well as regulating the expression of RNAKL, BMP-2, OPG	(19)
Icariin	*Epimedium brevicornum*		Activated the BMP signaling pathway and downregulated CTGF expression	(70)
N/A	BJTW	D-gal-induced aging model	Alleviated bone loss by modulating the levels of ALP, OCN, OPG, and RANKL	(75)
N/A	*Fructus Ligustri Lucidi*		Reversed the decrease of interleukin-2, tumor necrosis factor-alpha, and oxidative stress in the serum	(17)
Cycloastragenol	*Astragalus membranaceus*		Decreased serum bone resorption marker (TRACP), increased bone strength, reduced OC number, and improved bone formation	(79)
Diosgenin	*Dioscorea rhizome*		Increased frame and femur volume and decreased porosity and frame density	(77)
Peptide−Calcium Chelate	Antler (*Cervus elaphus*) Bone		Ameliorated the bone microstructure and alleviated age-related bone loss by increasing calcium absorption	(78)
Canthaxanthin	N/A		Increased the BMD, parameters of structural mechanics and biomechanics, bone calcium	(76)
N/A	ZWG	BMSCs	Regulated Wnt signaling and suppressed the expression p53, p21, and p16 to enhance cell proliferation	(92)
N/A	*Astragalus membranaceus*		Regulated the VD-FGF23-Klotho pathway, reversed the decreased cell viability and osteogenic ability	(105)
Astragalus Polysaccharide	*Astragalus membranaceus*		Increased Nanog, Sox2, and Oct4 expression, and impeded mitochondrial ROS accumulation	(87)
Ginkgolide B	*Ginkgo biloba*		Promoted osteogenesis and inhibited osteoclastogenesis by reducing ROS	(86)
Resveratrol	*Veratrum grandiflorum*		Activated AMPK and down-regulated ROS production	(85)
Catalpol	*Rehmannia glutinosa*		Reduced the percentage of senescent cells and enhanced the osteogenic differentiation	(103)
Tanshinone IIA	*Salvia miltiorrhiza*		Restored the cellular stemness and improved the aging state	(104)
Resveratrol and Anthocyanins	N/A	OB	Reduced *p53* mRNA expression and reduced RANKL-induced bone resorption	(99)
Echinacoside	*Herba Cistanches*		Inhibited p53 expression	(101)
N/A	DHJST	hBMSCs	Increased *RUNX2* and *BMP-2* gene expression, decreased the level of senescence	(102)
Resveratrol	*Veratrum grandiflorum*		Decreased the secretion levels of SASP and the expression levels of p53, p16, p21	(53)
*Humulus lupulus* Extract	*Humulus lupulus*	MC3T3-E1 cells	Alleviated oxidative stress and regulated bone metabolism	(88)

## Author contributions

YZ: Writing – original draft. XY: Writing – original draft. CZ: Writing – original draft. KF: Data curation, Formal analysis, Writing – review & editing. HL: Conceptualization, Data curation, Formal analysis, Supervision, Writing – review & editing. CW: Conceptualization, Funding acquisition, Supervision, Writing – review & editing.

## Glossary

OP: Osteoporosis
SOP: Senile osteoporosis
PMOP: Postmenopausal osteoporosis
OB: Osteoblast
OC: Osteoclast
BMSCs: Bone marrow mesenchymal stem cells
TCM: Traditional Chinese medicine
SASP: Senescence-associated secretory phenotype
ROS: Reactive oxygen species
D-gal: D-galactose
SAMP: Senescence-accelerated mouse/pron
EF: *Epimedium leaf*
FLL: *Fructus Ligustri Lucidi*
XZGB: Xianzhengubao
FTZ: Fufang Zhenzhu Tiaozhi
LEP: Leptin
QED: Qing’e Decoction
OA: Oleanolic Acid
EUL: *Eucommia ulmoides* leaf water extract
LB: *Lactobacillus bulgaricus*
GM: Gut microbiota
AM: *Astragalus membranaceus*
BGSSD: Bu-Gu-Sheng-Sui decoction
ACAE: Antrodia camphorata alcohol extract
OG: Orcinol glucoside
CTGF: Connective tissue growth factor
BJTW: Bajitianwan
GB: Ginkgolide B
AP: Astragalus Polysaccharide
Aβ: β-amyloid
HLE: *Humulus lupulus* L. Extract
SJZT: Si Jun Zi Tang
DHJST: Du-Huo-Ji-Sheng-Tang
hMSCs: Human mesenchymal cells
TSNA: Tanshinone IIA
